# Barriers to and Facilitators of Digital Health Technology Adoption Among Older Adults With Chronic Diseases: Updated Systematic Review

**DOI:** 10.2196/80000

**Published:** 2025-09-11

**Authors:** Jennifer Hepburn, Lynn Williams, Lisa McCann

**Affiliations:** 1 Department of Psychological Sciences and Health University of Strathclyde Glasgow United Kingdom; 2 Department of Computer and Information Sciences University of Strathclyde Glasgow United Kingdom

**Keywords:** older adults, chronic disease, digital health, technology adoption, rural health, co-design

## Abstract

**Background:**

Older adults with chronic diseases are key beneficiaries of digital health technologies, yet adoption remains inconsistent, particularly in rural areas and among certain demographic groups, such as older women.

**Objective:**

This systematic review aimed to identify barriers to and facilitators of digital health adoption among older adults with chronic diseases, with particular attention to rural-urban differences, co-design, and equity-relevant factors.

**Methods:**

This updated review built on a previously published review by extending the search to include PsycArticles, Scopus, Web of Science, and PubMed databases for studies published between April 2022 and September 2024. Gray literature from August 2021 onward was also included. Studies were eligible if they reported barriers to or facilitators of digital health adoption among adults aged ≥60 years with chronic diseases. Findings were mapped to the capability, opportunity, and motivation–behavior model and analyzed using the PROGRESS-Plus (place of residence; race, ethnicity, culture, and language; occupation; gender and sex; religion; education; socioeconomic status; and social capital–plus) equity framework. Quality was assessed using the Mixed Methods Appraisal Tool, and all results are reported in line with the PRISMA (Preferred Reporting Items for Systematic Reviews and Meta-Analyses) guidelines.

**Results:**

In total, 12 studies from the original review were retained, with 17 new peer-reviewed studies added, yielding a total of 29 studies in addition to 30 documents identified in the gray literature search. Barriers included limited digital literacy and physical and cognitive challenges (capability); infrastructural deficits and usability challenges (opportunity); and privacy concerns, mistrust, and high satisfaction with existing care (motivation). Facilitators included tailored training and accessible design (capability), health care provider endorsement and hybrid care models (opportunity), and recognition of digital health benefits (motivation). Health care providers emerged as both facilitators and barriers, positively influencing adoption when engaged and trained but hindering it when lacking confidence or involvement. Comparative analysis of rural and urban contexts was limited by inconsistent reporting of equity-relevant variables. However, gray literature suggested that rural users face additional infrastructural challenges but express higher satisfaction with local care, potentially reducing motivation for digital uptake. Gender differences were observed in 5% (3/59) of the peer-reviewed studies and gray literature sources, with older women showing lower adoption and differing outcome priorities. Co-design enhanced adoption, especially when involving not just older adults but also health care providers and community stakeholders.

**Conclusions:**

Digital health adoption among older adults is shaped by capability, opportunity, and motivation factors. Effective and equitable digital health strategies must address infrastructural and literacy barriers, engage health care providers through training and co-design, and ensure multistakeholder involvement. This review highlights that greater attention to standardized reporting of demographic variables, especially gender and rurality, is essential in digital health research to support inclusive implementation.

**Trial Registration:**

PROSPERO International Prospective Register of Systematic Reviews CRD42024586893; https://www.crd.york.ac.uk/PROSPERO/view/CRD42024586893

**International Registered Report Identifier (IRRID):**

RR2-https://doi.org/10.3399/bjgp25X742161

## Introduction

### Background

The global population aged ≥60 years is projected to double between 2015 and 2050, placing increasing pressure on health care systems [1,2]. Population aging is widely recognized as one of the most pressing challenges facing health and social care worldwide [3]. Chronic disease is the leading cause of death worldwide, posing a major public health burden [4]. Ischemic heart disease, stroke, and chronic obstructive pulmonary disease (COPD) are among the top causes of mortality alongside Alzheimer disease, lung cancer, diabetes, chronic kidney disease, and cirrhosis [5]. These conditions typically require ongoing long-term management and monitoring, such as blood glucose tracking for diabetes and blood pressure tracking for cardiovascular disease. Multimorbidity (more than one chronic disease) is rising sharply, with an estimated two-thirds of adults aged ≥65 years expected to live with multiple chronic conditions by 2035 [6,7]. Rural and remote areas are disproportionately affected, where health systems often face additional challenges due to dispersed populations and resource constraints [8,9].

To address these pressures, innovative strategies are needed to enhance chronic disease management, particularly in settings where health care access is limited. Digital health technologies (DHTs), including telemedicine, mobile apps, remote monitoring devices, and electronic health records, offer promising tools to support health care delivery, improve health outcomes, and enhance system efficiency [10,11]. The global uptake of digital health accelerated during the COVID-19 pandemic, when remote monitoring and web-based consultations became critical [12-14]. When implemented effectively, digital health solutions have the potential to reduce clinician workload [15]; improve patient safety and care quality [16]; and decrease the need for in-person visits, which is an especially valuable benefit in rural areas, where patients often travel long distances to access health care [17]. Despite these potential benefits, there is evidence suggesting that older adults and those in rural areas are less likely to engage with DHTs compared to their urban counterparts [18-20]. Paradoxically, while these groups stand to benefit the most, they often remain digitally excluded.

The importance of digital health is underscored by the World Health Organization (WHO) *Global Strategy on Digital Health 2020-2025*, which promotes digital solutions to enhance health system resilience and equity [11]. Similarly, the regional digital health action plan for the WHO European Region 2023 to 2030 highlights the need to build digital capacity across the public and health care workforce [21]. These priorities have stimulated research into barriers to and facilitators of digital health adoption, with recurring themes such as infrastructural challenges, digital literacy gaps, and the critical role of health care providers in supporting uptake [22].

Although a growing body of literature explores digital health adoption, most studies focus on general populations or specific diseases, with limited attention to older adults with chronic conditions, a group at heightened risk of digital exclusion. For example, individuals aged ≥65 years are 18 times more likely never to have used a health app compared to those aged 18 to 24 years [23]. Despite increasing internet use during the pandemic, 34% of adults aged ≥75 years remain offline, and 13% of those aged ≥65 years do not own a mobile phone [24]. According to a report by Age UK, limited digital skills remain a significant barrier, with an estimated 4.7 million people aged ≥65 years in the United Kingdom lacking the basic digital skills required to use the internet effectively. In addition, the report found that 49% of people aged ≥50 years find it difficult to access their general practitioners (GPs) due to challenges with digital or telephone triage systems. These barriers contribute to feelings of frustration, disempowerment, and disengagement among older adults [25]. It is important to note that older adults are a diverse group whose digital engagement is shaped by the intersection of multiple factors, such as age, gender, socioeconomic status, educational level, place of residence, and health status, which can combine to create unique patterns of digital inclusion or exclusion. Understanding the common barriers to and facilitators of digital health adoption in this population is critical to inform more inclusive digital health strategies. To support this focus on equity, the PROGRESS-Plus (place of residence; race, ethnicity, culture, and language; occupation; gender and sex; religion; education; socioeconomic status; and social capital–plus) framework was selected to guide both the rationale for this review and the structured extraction of equity-related variables during analysis. PROGRESS-Plus is an equity-oriented framework that also considers the *Plus* domains of age, disability, and other contextual factors. This framework is increasingly used in systematic reviews to assess whether and how health interventions account for social determinants of health, ensuring that findings support equitable access and outcomes across diverse populations [26].

Gender is one such factor, with studies suggesting that older women, particularly those in low-income settings, are less likely to adopt DHTs than men due to lower digital confidence, greater privacy concerns, and different expectations regarding the purpose of DHTs [27-29]. These differences underscore the importance of including gender in evaluations of digital health access. Recent reviews have highlighted persistent inequities in digital health adoption; for example, a scoping review of the WHO European Region found that digital health interventions were more often adopted by White, English-speaking, urban, and economically advantaged individuals, with limited evidence on underserved groups such as rural populations or ethnic minority groups [30]. Similarly, a review of digital health use during the COVID-19 pandemic found that rapid implementation sometimes exacerbated disparities, particularly among those with limited access to devices or low digital literacy [31]. These findings underscore the importance of applying equity frameworks such as PROGRESS-Plus when evaluating digital health uptake.

### Previous Work

A previous scoping review that investigated barriers to and facilitators of older adults using eHealth identified common barriers such as lack of self-efficacy, insufficient knowledge and support, concerns over functionality, and lack of clear information about the benefits of digital health [32]. However, few reviews have focused specifically on older adults with chronic diseases, with the notable exception of a systematic review by Bertolazzi et al [33], which explored the barriers and facilitators influencing adoption of DHTs by older adults with chronic diseases. The review identified 5 domains influencing adoption: demographic and socioeconomic factors, health-related factors, dispositional attitudes, technology-related barriers, and social influences. The key facilitators were perceived usefulness, early introduction of technology, social support, and user-friendly design. Barriers included digital literacy, technical issues, a preference for existing ways of managing health, and lack of support.

### Goal of This Study

This review significantly extended the work by Bertolazzi et al [33] in both scope and depth to capture a rapidly evolving digital health landscape. While the original review included studies up to April 2022, it missed a critical wave of literature emerging during and after the COVID-19 pandemic, a period marked by a surge in the deployment and evaluation of DHTs [34,35]. By identifying studies published between April 2022 and September 2024, this review broadened the evidence base to reflect more recent trends, challenges, and innovations in digital health adoption among older adults with chronic diseases.

In addition to updating the time frame, this review addresses key limitations of the earlier work by incorporating gray literature, an important yet often overlooked source in fast-moving fields such as digital health. Government reports, policy briefs, and non-peer-reviewed evaluations provide timely, real-world insights. This is particularly relevant to equity, as published literature on digital health often underrepresents older adults [36] and specifically older adults in rural communities [37]. As Landerdahl Stridsberg et al [38] note, “Gray literature is particularly relevant for digital health and HWT… Systematic reviews… should consider including gray literature based on a systematic approach.” This approach is consistent with PRISMA (Preferred Reporting Items for Systematic Reviews and Meta-Analyses) 2020 guidance [39], which recognises gray literature as a valid evidence source in systematic reviews where eligibility criteria are applied consistently and search processes are transparent.

This review also deepened the analysis by examining previously underexplored dimensions. While the original review noted the value of involving older adults in design, it did not assess the use or impact of co-design approaches. We addressed this by extracting data on co-design and its influence on adoption. Similarly, rurality, a key determinant of access and equity in digital health, was not systematically analyzed. Where reported, we extracted rural-urban comparisons to better understand how context shapes adoption. Equity-related reporting itself also varied widely in the previous review. To address this, we applied the PROGRESS-Plus framework to assess how studies accounted for equity-relevant factors such as gender, geography, and socioeconomic status, dimensions that are essential for informing inclusive digital health strategies.

Finally, we applied a theoretical lens absent from the original work. Using the capability, opportunity, and motivation–behavior (COM-B) model [40], we categorized barriers and facilitators to provide a more structured, behaviorally informed interpretation of the evidence. This theoretical grounding enhances the potential for actionable insights to inform intervention design and policy. This review is an update of the review by Bertolazzi et al [33] and applied the same inclusion criteria, with the search extended from April 2022 to September 2024. Limitations identified in the original review informed the scope and methods of this update, including the addition of gray literature and attention to rurality, co-design, and equity considerations.

### Objectives

This review aimed to provide a more comprehensive understanding of the factors that influence digital health uptake among older adults with chronic diseases. The review was guided by the following questions:

1. What are the main factors hindering the adoption of DHTs by older adults with chronic diseases?
2. What are the main factors facilitating the adoption of these technologies?
3. How do these barriers and facilitators vary across equity-relevant factors such as place of residence (urban or rural) and gender?
4. In what ways does co-design support the adoption of DHTs for chronic disease management?

## Methods

### Protocol and Registration

This review followed a prespecified protocol, which was registered with PROSPERO (CRD42024586893).

### Overview

This study is an updated systematic review that synthesizes research results on the barriers to and facilitators of the uptake of DHTs by older adults with chronic diseases following the PRISMA guidelines (see the PRISMA checklist in Multimedia Appendix 1) [39]. To address specific limitations identified in the original review, the update expanded the search to include gray literature, increased attention to rural contexts, and incorporated additional data extractions on co‑design processes. The full search strategies from Bertolazzi et al [33] were available and retained for reference. All studies included in the original review were rescreened using the same inclusion criteria as this update, with only eligible studies retained. In accordance with the PRISMA 2020 guidance for updated reviews [39], we searched only from April 2022 to September 2024 as the previous review had already conducted a comprehensive search from 2012 to April 2022.

### Information Sources and Search Strategy

Four electronic databases were searched: PsycArticles, Scopus, Web of Science, and PubMed. Searches were conducted for publications dated April 2022 to September 2024 using a Boolean strategy combining keywords and MeSH (Medical Subject Headings) based on the population, intervention, comparison, and outcome framework [41] (Textbox 1; see Multimedia Appendix 2 for the search strategy). The date restriction was intentional as the period from 2012 to April 2022 had already been searched in the original review and rerunning it risked unnecessary duplication without yielding additional eligible studies.

**Search terms using the population, intervention, comparison, and outcome mnemonic.**

**Population**

“elderly” OR “older adult” OR “ageing people”

**Phenomena of interest**

“technology” OR “gerontechnology”

**Context**

“chronic disease” OR “chronic illness” OR “long-term conditions” OR “chronic conditions”

### Eligibility Criteria

The eligibility criteria used in the original review for the study selection process are presented in Textbox 2 [33].

The inclusion and exclusion criteria used in this review match those in Textbox 2 with the following amendment: studies were included if they were published between April 2022 and September 2024. Although the database searches only included empirical studies, the gray literature search included nonempirical publications. In line with the original review by Bertolazzi et al [33], *chronic disease* was defined as in their review: “health problems that are long lasting, generally progressive, and require ongoing management over a period of years or decades.” This definition includes but is not limited to cardiovascular diseases (eg, hypertension and heart failure), respiratory diseases (eg, COPD and asthma), diabetes and other endocrine disorders, neurological conditions (eg, dementia and Parkinson disease), chronic kidney disease, and cancer (where ongoing management is required). For inclusion in this review, study participants had to be older adults (aged ≥60 years) with one or more diagnosed chronic diseases as defined previously.

**Inclusion and exclusion criteria used in the original review.**

**Inclusion criteria**

- Population: participants aged ≥60 years and diagnosed with one or more chronic diseases
- Phenomenon of interest: focus on barriers to or facilitators of the adoption of digital health technologies for chronic disease management and technology targeting older adults
- Context: empirical studies (qualitative, quantitative, or mixed methods) and published in English between 2012 and 2022

**Exclusion criteria**

- Population: mixed-age samples (including those aged <60 years)
- Phenomenon of interest: studies evaluating a general health care service without focus on the specific enabling digital tools
- Context: nonempirical publications (eg, reviews, editorials, dissertations, conference abstracts, or narrative-only studies)

### Searching for Gray Literature Studies

A targeted gray literature search was conducted between August 2024 and January 2025. This focused on identifying white papers, policy documents, and guidelines related to digital health implementation in rural settings for older adults with chronic conditions. To reflect developments since the publication of the WHO “Global strategy on digital health 2020-2025” [11], only documents published from August 2021 onward were included.

The gray literature search included manual searches of international government websites and research organizations and targeted searches for dissertations and conference proceedings. A total of 30 gray literature documents were included. These were not formally quality appraised or counted among the included studies but were used to contextualize findings, particularly for rural settings.

### Study Selection

Records were initially screened by title and abstract followed by full‑text assessment for eligibility. Two reviewers independently screened titles and abstracts and then full texts for eligibility, with discrepancies resolved through consensus or a third reviewer. Duplicates were removed before screening. All studies from the original review were rescreened in full against the inclusion criteria for this update. Only those meeting the criteria were retained for synthesis alongside newly identified studies from the extended search period.

### Data Extraction

Data were extracted using a data extraction tool within Covidence (Veritas Health Innovation) by 2 independent reviewers. Extracted data included (1) barriers to and facilitators of digital health adoption (outcomes); (2) age, gender, chronic conditions, urban or rural residence, and other equity variables relevant to the PROGRESS-Plus framework (participant characteristics); (3) type of DHTs (eg, telemedicine, mobile app, or wearable), setting, and whether co-design was reported (intervention characteristics); and (4) study design (qualitative, quantitative, or mixed methods), country, sample size, and publication year (study characteristics).

Where data were unclear or missing, this was noted in the extraction tables, and no assumptions were made.

### Theoretical Framework for Data Interpretation and Equity Considerations

This review applied the COM-B framework [40] to interpret and map research findings on the barriers and facilitators influencing older adults with chronic diseases in their use of digital technology for self-management. As noted in the Introduction section, the PROGRESS-Plus framework was also applied to identify equity-relevant factors reported across the studies in line with Cochrane recommendations [26]. This approach enabled both behavioral and equity-focused interpretations of the findings.

### Quality Assessment

The methodological quality of the included studies was assessed using the Mixed Methods Appraisal Tool (MMAT) [42]. Two reviewers independently conducted the quality assessment, with discrepancies resolved through discussion or consultation with a third reviewer.

### Synthesis Methods

Due to heterogeneity in study designs, interventions, and reporting formats, a narrative synthesis was conducted. Extracted data were manually tabulated using Covidence and Microsoft Excel, and thematic analysis was applied to synthesize findings within the COM-B framework.

1. Eligibility for synthesis: all the included studies were eligible for narrative synthesis and were grouped by study design and type of digital health intervention.
2. Exploring heterogeneity: heterogeneity was explored narratively, with attention to differences across rural versus urban populations, intervention types, and study contexts.
3. Sensitivity analyses: no sensitivity analyses were conducted as no meta-analysis was performed and no pooled effect estimates were produced.
4. Risk-of-bias assessment: no formal assessment of publication bias was conducted as no meta-analysis was performed.
5. Certainty assessment: the certainty of evidence was not formally graded (eg, using the Grading of Recommendations Assessment, Development, and Evaluation) as the synthesis focused on qualitative and mixed methods findings.

Gray literature was presented separately from peer‑reviewed studies to maintain transparency as these sources were not subject to the same methodological quality appraisal. This separation allows readers to interpret peer‑reviewed evidence independently while still considering the contextual value of gray literature, which is integrated into the Discussion section.

## Results

The findings presented in this section are from the updated review and include both newly identified studies and eligible studies retained from the original review following rescreening against the inclusion criteria. A brief comparison with the original review is provided in the Discussion section.

### Identification of Studies

**Figure 1:**
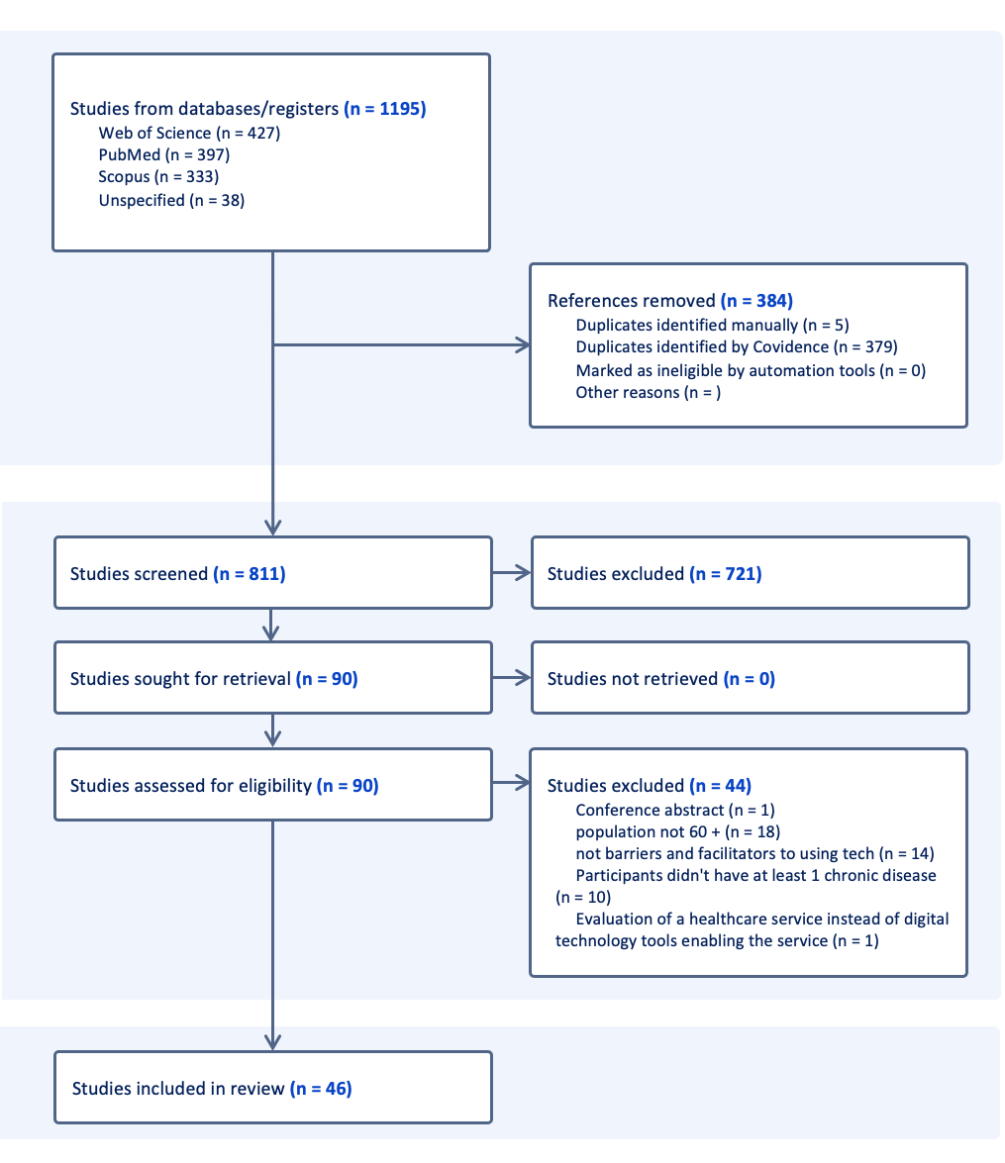
PRISMA (Preferred Reporting Items for Systematic Reviews and Meta-Analyses) flowchart.

A total of 1963 records were retrieved across databases. After removing duplicates, of the 1963 records, 811 (41.31%) were screened. Two authors independently screened titles and abstracts followed by full-text assessment against the eligibility criteria, with discrepancies discussed with a third reviewer. As shown in the PRISMA flowchart (Figure 1), 46 studies were initially included, comprising 17 (37%) new studies published between April 2022 and September 2024. During the data extraction process, eligibility issues in several studies included in the previous review by Bertolazzi et al [33] were identified. Specifically, 59% (17/29) of the studies previously included did not meet the stated inclusion criteria upon re-evaluation and, therefore, were excluded from this review. This explains the difference between the 46 studies initially shown in the PRISMA diagram and the 29 peer-reviewed studies included in the final synthesis. Exclusion was based on one or more of the following factors: inclusion of mixed-age samples that did not isolate findings for adults aged ≥60 years, digital technologies not specifically targeting older adults, or the papers not including barriers to or facilitators of the adoption of DHTs for chronic disease management.

The 12 studies that did meet the criteria were retained and included alongside 17 newly identified studies. As a result, this updated review comprised a total of 29 peer-reviewed studies. Full details on the excluded studies and the reasons for their exclusion are provided in Multimedia Appendix 3 [43-60]. The PRISMA diagram reflects the peer-reviewed studies only. Gray literature was identified and screened separately, with results reported in a dedicated section of this manuscript.

### Characteristics of the Included Studies

A summary of study characteristics is provided in Multimedia Appendix 4 [61-89].

Among the 29 included studies, study designs comprised 1 (3%) randomized controlled trial [61] and 5 (17%) mixed methods studies [62-66]. Most studies were qualitative**―**38% (11/29) were interview-based studies [67-77], and 10% (3/29) used focus groups [78-80]. In total, 31% (9/29) of the studies used quantitative designs, including questionnaires and surveys [81-89].

The type of technologies used for managing chronic diseases varied widely, including wearable devices [67,72,74,77,89], mobile apps [61,68,70,73,85], digital health platforms [62,64,69,79], patient portals [80], telemonitoring [63,66,75], telemedicine [71,81,83], telehealth [76,87], information and communications technology services [78,84,86], assistive robots [65], and active video games [74].

Regarding the type and number of chronic diseases addressed, most studies (17/29, 59%) focused on multiple conditions [61-64,66,68,72,75,78,80-82,84,85,87-89]. A total of 24% (7/29) of the studies examined a single, specific chronic condition**―**cognitive impairment [65], COPD [67,70,74,76], or heart failure [73,77].

### Barriers and Facilitators (COM-B Framework)

After summarizing the study characteristics, the barriers and facilitators were grouped under the COM-B domains: capability, opportunity, and motivation (see the list of barriers and facilitators in Multimedia Appendix 5 [61-118]).

#### Capability

Limited digital literacy [71,77,83-85,89] and lower educational attainment [74,76,85,86] were the most common barriers. Other barriers included unfamiliarity with smartphones [78] or the internet [69]; low health literacy [79]; early difficulties with the technology [75,77,79]; being acutely unwell at the time of installation [75]; and age-related visual, manual dexterity, or memory challenges [62,66,67,75,76,83].

Facilitators in this domain included the provision of clear instructions, structured training sessions, and technical support [63,64,68,70,71,75,77,79,80,82,85,87], as well as pretraining initiatives aimed at building users’ technical skills before engaging with the technology [79]. Technology that was simple and easy to use [63,65,72,82] and early integration of the technology into care routines [65] further supported engagement.

#### Opportunity

Key barriers within the opportunity domain were primarily structural and contextual. This included limited access to digital devices [72,85], high technology costs [63,77-79], poor internet connectivity and technical issues [62,75,82,87], lack of goal personalization [68], single-condition focus [82], reduced social interaction [76,78], and reliance on family for support [69,70].

Practical and emotional support from family, friends, and caregivers promoted uptake [63,67,68,70,71,75,79,80,87], with involvement from adult children identified as particularly influential [67,76]. Co-design was reported in 24% (7/29) of the included studies [62-64,68,79,82,85], and person-centered approaches to co-design were specifically highlighted as facilitators in 29% (2/7) of these studies [82,85]. Related findings specific to health care providers are presented in the Health Care Providers section.

#### Motivation

Many older adults reported apprehension about using DHTs, often driven by concerns about data privacy [67,70,76] and mistrust of digital systems [85]. Emotional responses such as fear, anxiety, and embarrassment were commonly reported when navigating unfamiliar technologies [67,77,80,85] alongside a fear of making mistakes [79]. For some, the technology acted as a distressing reminder of illness or the possibility of recurrence [72,75,79], and negative emotions were triggered when health goals were not achieved or when data readings indicated concerning results [68,79]. Others viewed digital tools as an added burden [64], questioning their reliability [64,67,75,76] or doubting their value or benefit to health [68,69,73]. Feelings of ambivalence [76], fears of losing autonomy [78,79], and a general lack of interest in technology [85] further reduced motivation. In some cases, social perceptions also played a role, with concerns that using digital health tools might make individuals appear older or more dependent [78].

Key motivational facilitators included understanding the health benefits of the technology, gaining insights into condition management, setting goals, and feeling empowered to take an active role in care [63,64,67-69,72-74,76,81]. For some, the technology provided reassurance and a sense of safety, particularly when it enabled ongoing remote monitoring by health care professionals [66,75,78]. Positive experiences with the technology, including successful use [76] and receiving personalized feedback [79], further strengthened motivation to engage. Related findings specific to health care providers are presented in the Health Care Providers section.

### Health Care Providers

This section aligns with the COM‑B framework, with subthemes mapping primarily to the opportunity and motivation domains. It is presented separately due to the prominence and frequency of health care provider influences across the included studies.

#### Opportunity

In some instances, health care providers themselves acted as barriers, particularly when they appeared disinterested or lacked time to engage with patient-generated data [64,73,78]. Uptake was also less likely if the technology had not been recommended by a trusted health care professional [72]. Conversely, endorsement or active encouragement from trusted clinicians increased engagement [63,75-77,79,118]. Digital services that strengthened continuity of care or improved communication with clinicians were more likely to be accepted [64,69,70,80].

#### Motivation

Concerns that digital technologies might reduce or replace personal interaction with health care providers were common, especially among older adults who valued in-person care [71,72,75]. The perception that remote consultations were inferior to face-to-face interactions further contributed to reluctance [70]. However, clinician engagement with patient-generated data was also reported to enhance patient motivation [68,74], and collaborative involvement in care planning fostered a sense of empowerment [63]. Trust in health care providers remained a critical motivational driver for digital health uptake [71].

### Rural and Urban Differences

A total of 14% (4/29) of the included studies reported on whether the study took place in a rural or urban area [66,72,75,88], and 75% (3/4) of these studies took place in rural areas. These studies identified the following: unreliable technology use in rural areas due to poor internet connectivity [75], no significant differences in telenursing needs between rural and urban participants regarding desired service features [88], and the fact that combining personalized telemonitoring with a case manager familiar with patients allowed for timely responses to alerts without overburdening GPs or specialists [66].

### Gray Literature: Rural-Specific Barriers and Facilitators

Gray literature findings are presented separately here for transparency but are synthesized with peer‑reviewed evidence in the Discussion section. Additional barriers and facilitators specific to rural contexts were identified in the gray literature that were not reported in the included peer-reviewed studies.

#### Capability

Barriers to capability included the absence of locally delivered and culturally tailored training programs [90-95]. Among health care professionals, a lack of dedicated training, ongoing support, and clear guidance on telehealth use contributed to slower adoption and reduced confidence [93,94,97]. As previously discussed, limited digital literacy among older adults emerged as a key barrier in the peer-reviewed studies, and this was reinforced by the gray literature sources [90,94,95,98-109]. In addition, health care professionals’ unfamiliarity with digital health tools and previous negative experiences further hindered engagement [96,97,107].

Facilitators included the provision of targeted local training and culturally relevant support materials tailored to rural communities [90,93,101]. These approaches were reported to enhance digital readiness and build confidence among both patients and clinicians [93].

#### Opportunity

Poor internet connectivity and fragmented infrastructure were consistently highlighted [90,91,96-102,106,107,109,111-113]. Additional challenges included limited promotion and awareness of available digital tools [102,113,114] alongside workforce constraints such as staff shortages, outdated or nonintegrated equipment, and inadequate resourcing [90,100,108,109,113].

Facilitators included investment in infrastructure, financial and logistical implementation support, and the use of low-technology or hybrid models such as telephone-based care to mitigate digital exclusion [100,115]. Involvement of trusted link workers, digital champions, community navigators, and clinicians was reported to support uptake and reduce burden on GPs [66,92,96,97].

#### Co-Design

Several sources also identified co-design as a facilitator to ensure that digital tools were relevant and usable in rural contexts. This included participatory approaches that involved older adults, families, caregivers, and community stakeholders to support adoption [90,97,104,107,110,114-117]. Barriers included the absence of participatory design [98], limited consideration of older adults’ cognitive and physical needs [106,110], and insufficient community involvement during development [115]. Several sources noted that the lack of family or community input contributed to resistance or reduced the relevance of DHTs [110,115,116].

#### Motivation

Gray literature identified several motivational barriers affecting digital health uptake in rural areas. These included mistrust of digital platforms [90,95,99,108,110], fear that technology would replace human interaction [101], and a preference for face-to-face care [101-103,114]. Cultural values such as self-sufficiency [118], feelings of underrepresentation in national policy [106], and mistrust of the government [110] were also reported. A lack of face-to-face contact with health care professionals was associated with reduced trust and satisfaction [108]. Culture and trust were described as influencing comfort and willingness to engage with digital tools [110]. In addition, one source reported a positive association between GP availability and satisfaction with local health care, suggesting that perceptions of adequate in-person care provision may reduce motivation to adopt digital alternatives [116].

### Equity and PROGRESS-Plus Findings

The PROGRESS-Plus analysis highlighted inconsistent reporting of equity-relevant factors. Characteristics such as race and ethnicity, socioeconomic status, and occupation were often underreported. Only 14% (4/29) of the studies provided data on place of residence (urban vs rural), and none offered a clear operational definition of rurality, limiting comparative analysis.

In total, 93% (27/29) of the studies reported on gender; however, only 11% (3/27) of these studies provided any comparative insights and identified gender as a factor influencing digital health adoption. Men were reported to have a stronger preference for staying up-to-date with technology [67], showed a preference for autonomy-enhancing services [78], and were less likely than women to report technology unreadiness [81]. Gray literature further highlighted that older women, particularly in low-income settings, were less likely to engage with DHTs [90,91,95,111].

This lack of data limited our ability to compare subgroups on many PROGRESS-Plus factors. Multimedia Appendix 6 [61-89] provides a list of these factors. These findings are consistent with broader evidence that reports that digital health adoption tends to favor younger, urban, and socioeconomically advantaged populations, whereas rural and minority groups remain underrepresented [31,32]. This highlights the need for more systematic equity-focused reporting in digital health research.

### Quality Assessment

The MMAT was used to assess the methodological quality of all the included peer-reviewed studies. No studies were excluded based on quality in line with guidance for mixed methods systematic reviews [26]. Qualitative studies generally achieved high MMAT scores (4 to 5 stars), with strengths including clearly defined questions, appropriate data collection, and detailed analysis. Only 2/14, (14%) [67,76] explicitly reported reflexivity with the remaining studies (12/14, 86%) [68-75,77-80] focusing on other strategies to enhance rigour, limiting transparency about researcher influence. Quantitative studies were of moderate to high quality (3 to 4 stars), typically showing appropriate methods and outcome measures. However, several had methodological limitations with 3/9 (33%) [83,84,89] employing unclear or convenience sampling approaches, 3/9 (33%) [82,83,88] failing to report or partially reported response rates and 6/9 (67%) [42,82-85,89] relying at least partially on unvalidated self-report measures. Mixed methods studies were generally of good quality. All 5/5 (100%) [62-66] integrated qualitative and quantitative strands effectively. The majority (4/5, 80%) [62-65] clearly justified their mixed methods design, and 1/5 (20%) [66] did not provide a rationale for combining methods. The full MMAT ratings for each study are provided in Multimedia Appendix 7 [61-89].

## Discussion

### Principal Findings

This systematic review synthesized evidence on the barriers to and facilitators of digital health adoption among older adults with chronic diseases using the COM-B model and PROGRESS-Plus framework. The main barriers to adoption were limited digital literacy and confidence (capability); poor internet access, high costs, and lack of integrated technical support (opportunity); and fears regarding privacy, mistrust, and satisfaction with existing services (motivation). Facilitators included user-friendly design and training (capability), trusted health care provider endorsement and hybrid care models (opportunity), and perceived benefits of digital health and positive user experiences (motivation).

Regarding rural and urban differences, a key insight from this review is the paradox that, although rural patients are physically distant from health care services and stand to benefit from digital health, they often report high satisfaction and trust in their existing local care. This may act as a motivational barrier, reducing perceived need to adopt digital tools. The gray literature identified a strong preference for face-to-face care [101-103,114]. This may be partly explained by the fact that rural regions often have better GP-to-patient ratios than urban areas, and strong patient-provider relationships may reinforce satisfaction with in-person care [116].

Gray literature sources identified additional challenges specific to rural settings. These included limited digital familiarity among health care professionals [96,107]; insufficient training and ongoing support [93,94]; and structural barriers such as workforce shortages, outdated equipment, and resource constraints [90,100,108,109,113]. One proposed solution to mitigate these issues is the introduction of case managers or community link workers who can engage older adults directly and help reduce the burden on overstretched health care providers [66,92,96,97].

This review also highlights gender as an underexplored factor not addressed in the original review [34]. Evidence from the gray literature indicated that older women, particularly in low-income settings, were less likely to engage with DHTs than men [90,91,95,111]. In addition, peer-reviewed studies suggested that men and women may differ in their preferences for how digital health services are delivered [67,78]. These findings underscore the importance of routinely reporting gender and other equity-related variables to prevent the design of one-size-fits-all digital health solutions. It is also important to acknowledge that sex and gender are frequently used interchangeably in the literature, including studies applying frameworks such as PROGRESS-Plus. This can potentially obscure important distinctions between biological sex and socially constructed gender roles and how they shape health behavior and digital access.

Regarding co-design, while the original review [33] acknowledged the value of involving older adults in design, this review extended that understanding by showing that involving health care providers and other stakeholders (eg, caregivers, local organizations, and community gatekeepers) in co-design processes is also essential in encouraging uptake [90,98,105,109,110,112,116,117,119]. This broader approach ensures that digital tools are not only user-friendly for patients but also practical for providers and sustainable within health care systems.

### Limitations

Limitations include inconsistent reporting of equity-relevant variables across peer-reviewed studies. Because many primary studies did not report key demographic details, our ability to analyze subgroup differences was constrained. The exclusion of non–English-language studies is also a potential limitation.

Studies reporting successful adoption might be more likely to be published, potentially biasing the literature; however, our inclusion of gray literature helped mitigate this to some extent. Although considerable care was taken in conducting the gray literature search, the manual systematic search may have led to the omission of some relevant documents. The variability in how gray literature is indexed and the lack of consistent search tools make comprehensive retrieval challenging despite targeted efforts. Nonetheless, by synthesizing multiple data sources and using established frameworks, we believe that the findings identified are reliable and relevant.

### Comparison With Previous Work

Our findings align with those of the original review [33] on general barriers such as digital literacy gaps, infrastructural barriers, and motivational challenges as key hurdles for older adults adopting digital health. This review added new insights on gender differences, rural satisfaction paradoxes, the dual role of health care providers, and the expanded role of multistakeholder co-design, broadening the evidence base.

### Future Research Directions

This review highlights the need for future studies to improve demographic reporting, particularly regarding characteristics such as rurality and gender, which are associated with digital exclusion. To improve the clarity of such research, future studies should report sex and gender as separate variables in line with the Sex and Gender Equity Research Guidelines [120]. Applying frameworks such as the COM-B and PROGRESS-Plus during the design and reporting could improve consistency and enable a more meaningful interpretation of barriers to and facilitators of DHT engagement. In addition, there is a need to adopt an intersectional approach that recognizes how multiple factors such as age, gender, and rurality shape individual experiences. This is essential for understanding who is most at risk of digital exclusion and for designing interventions that address the complex and intersecting inequalities facing older adults. Finally, the development and application of equity indicators for digital health interventions would ensure that equity considerations are systematically embedded in both research and implementation.

### Conclusions

This review confirms that digital health adoption among older adults with chronic diseases is shaped by a complex mix of capability, opportunity, and motivation factors. While digital health offers potential to improve access and outcomes, high satisfaction with existing care (especially in rural areas) and provider-related barriers highlight the need for targeted strategies. Embedding multistakeholder co-design and paying close attention to equity considerations such as gender and rurality in reporting and design are likely to be critical to developing digital health solutions that are inclusive, effective, and sustainable.

## Appendix

Multimedia Appendix 1 PRISMA (Preferred Reporting Items for Systematic Reviews and Meta-Analyses) checklist.

Multimedia Appendix 2 Search strategy.

Multimedia Appendix 3 Excluded studies.

Multimedia Appendix 4 Study characteristics.

Multimedia Appendix 5 Barriers and facilitators.

Multimedia Appendix 6 Mixed Methods Appraisal Tool results.

Multimedia Appendix 7 PROGRESS-Plus (place of residence; race, ethnicity, culture, and language; occupation; gender and sex; religion; education; socioeconomic status; and social capital–Plus) variable extraction.

## Abbreviations

COM-B: capability, opportunity, and motivation–behavior
COPD: chronic obstructive pulmonary disease
DHT: digital health technology
GP: general practitioner
MeSH: Medical Subject Headings
MMAT: Mixed Methods Appraisal Tool
PRISMA: Preferred Reporting Items for Systematic Reviews and Meta-Analyses
PROGRESS-Plus: place of residence; race, ethnicity, culture, and language; occupation; gender and sex; religion; education; socioeconomic status; and social capital–plus (“plus” includes additional factors such as age and disability)
WHO: World Health Organization
