# Supplementary material for: Barriers to and Facilitators of Digital Health Technology Adoption Among Older Adults With Chronic Diseases: Updated Systematic Review
Source: JMIR Aging. 2025 Sep 11;8:e80000. doi: 10.2196/80000 (PMC12464506; doi:10.2196/80000)
Supplement: Multimedia Appendix 2 [file aging_v8i1e80000_app2.docx]

**Original Search Strategy (Bertolazzi et al., 2024)**

| Database | PubMed, Scopus, PsycArticles, Web of Science |
| --- | --- |
| Search strategy | #1: elderly OR aged OR older OR older adult* OR frail OR frail older OR frail older OR frail older adult* OR elder OR frail elder* OR geriatric OR elderly people OR old people OR senior OR aging people OR ageing people  #2: technology OR gerontechnology  #3: chronic disease OR chronic illness OR long-term conditions OR chronic conditions  #4: English[language]  #5: ("2012"[Date - Publication]: "2022"[Date - Publication])  #6: #1 AND #2 AND #3 AND #4 AND #5 |

**Updated Search Strategy for Current Review**

Searches were conducted across four databases: **PubMed, Scopus, Web of Science, and APA PsycArticles**, using a combination of MeSH terms (PubMed only) and free-text keywords adapted for each platform. Searches were limited to **English-language publications** between **April 2022 and September 2024**.

Note: Field tags such as TI (title), AB (abstract), and MH (MeSH) were applied where supported. The search was tailored to the syntax of each database.

**Final search conducted:** September 20, 2024

**Search Concept Blocks**

1. **Population Terms**

- Keywords: older adults, older people, elderly, seniors, aged 60+, geriatric, ageing
- MeSH Terms (PubMed): Aged, Aging, Aged 80 and Over, Gerontologic Care

1. **Chronic Conditions**

- Keywords: chronic disease, chronic illness, long-term conditions, multimorbidity, diabetes, cardiovascular disease, cancer, stroke, COPD
- MeSH Terms: Chronic Disease, Cardiovascular Diseases, Diabetes Mellitus, Neoplasms

1. **Digital Health Technologies**

- Keywords: digital health, eHealth, telehealth, mHealth, mobile apps, wearable technology, remote monitoring, patient portal, assistive technology, smartwatch
- MeSH Terms: Telemedicine, Mobile Applications, Remote Consultation, Digital Technology

1. **Adoption and Implementation**

- Keywords: adoption, acceptability, barriers, facilitators, uptake, engagement, implementation, user experience, digital inclusion
- MeSH Terms: Health Services Accessibility, Patient Acceptance of Health Care

**Example Full PubMed Search String**

("Aged"[Mesh] OR "Aged, 80 and over"[Mesh] OR "older adult*"[tiab] OR "elderly"[tiab] OR "seniors"[tiab] OR "geriatric*"[tiab]) AND ("Chronic Disease"[Mesh] OR "multimorbidity"[tiab] OR "chronic illness"[tiab] OR "long-term condition*"[tiab] OR "diabetes"[tiab] OR "cardiovascular"[tiab] OR "cancer"[tiab]) AND ("Telemedicine"[Mesh] OR "Mobile Applications"[Mesh] OR "eHealth"[tiab] OR "digital health"[tiab] OR "telehealth"[tiab] OR "remote monitoring"[tiab] OR "wearable technology"[tiab]) AND ("adoption"[tiab] OR "acceptability"[tiab] OR "barrier*"[tiab] OR "facilitator*"[tiab] OR "implementation"[tiab] OR "uptake"[tiab]) AND (English[lang]) AND ("2012/01/01"[Date - Publication] : "2024/09/30"[Date - Publication])

**Search Concept Summary Table**

| **Concept** | **Keywords** | **MeSH Terms** |
| --- | --- | --- |
| Population | older adults, elderly, seniors, aged 60+, geriatric, ageing | Aged, Aging, Aged 80 and Over, Gerontologic Care |
| Conditions | chronic disease, multimorbidity, long-term conditions, diabetes, cancer, stroke | Chronic Disease, Cardiovascular Diseases, Diabetes Mellitus, Neoplasms |
| Technologies | digital health, eHealth, mHealth, telehealth, wearable technology, smartwatch, patient portal | Telemedicine, Mobile Applications, Remote Consultation, Digital Technology |
| Implementation | adoption, barriers, facilitators, uptake, engagement, acceptability, digital inclusion, user experience | Health Services Accessibility, Patient Acceptance of Health Care |

This search strategy was used to identify peer-reviewed studies on the barriers and facilitators to digital health technology adoption among older adults with chronic conditions. Additional grey literature sources were identified using targeted keyword searches on relevant organisational websites.
