# Supplementary material for: Barriers to and Facilitators of Digital Health Technology Adoption Among Older Adults With Chronic Diseases: Updated Systematic Review
Source: JMIR Aging. 2025 Sep 11;8:e80000. doi: 10.2196/80000 (PMC12464506; doi:10.2196/80000)
Supplement: Multimedia Appendix 3 [file aging_v8i1e80000_app3.docx]

# Papers included the Bertolazzi et al (2024) review which do not meet the inclusion exclusion criteria

The aim of this review is to update and extend the original systematic review carried out by Bertolazzi et al (2024) and provide an up-to-date and more nuanced understanding of the literature. We will use the same inclusion and exclusion criteria as Bertolazzi et al (2024) (see Table 1) and this will be applied to papers published since April 2022. It was our intention to add these to the existing papers for data extraction. However, during the data extraction process it became apparent that several of the existing papers identified by Bertolazzi et al (2024) do not meet the inclusion criteria and have therefore been excluded from our review.

Table 1: Bertolazzi et al. (2024) Inclusion and Exclusion Criteria

| **Inclusion Criteria** | **Exclusion Criteria** |
| --- | --- |
| The sample must include participants aged 60 or over | Mixed sample population with participants above and below 60 years of age |
| The studies must focus on facilitators or barriers to the adoption of technologies related to chronic disease management | Studies that evaluated a healthcare service instead of digital technology tools enabling the service (e.g. studies that evaluated the general telemedicine service without focusing on the platform enabling telemedicine services). |
| The studies must be empirical and use qualitative, quantitative or mixed methods | Publications such as theoretical contributions, letters to the editor, systematic or scoping reviews, dissertations, conference proceedings or those adopting non-standardised techniques and lacking sufficient analytical rigour |
| The studies must be published in English |  |
| The studies must focus on technology targeting older people |  |
| The sample must include participants affected by chronic disease |  |

| **Study** | **Participants** | **Chronic Disease** | **Focus** | **Type of Study** | **Target Population** | **Exclusion Criteria** |
| --- | --- | --- | --- | --- | --- | --- |
| AlMahadin et al. (2020) [104] | 12 participants with an age range of 56-88  4 healthcare professionals aged 52-61 | Parkinson’s Disease | Barriers and facilitators to the adoption of wearable devices to assess motor symptoms for Parkinson’s Disease. | Qualitative (focus groups) | Older adults | Mixed sample of adults aged above and under 60. |
| Ancker et al. (2015) [105] | 22 participants with an age range of 37-89.  7 healthcare professionals. | Multiple chronic conditions | Barriers and facilitators to digital health technology to track health conditions through diet and exercise apps or blood glucose meters. | Qualitative (interviews) | People with chronic diseases | Mixed sample of adults aged above and under 60.  The focus was not on technology targeting older people. |
| Banbury et al. (2014) [106] | 52 participants with an age range of 49-93 | Multiple chronic conditions | The acceptability of home-based group education via videoconferencing for older people with chronic disease. | Qualitative (interviews, focus groups and a journal detailing the technology implementation) | Older adults | Mixed sample of adults aged above and under 60. |
| Christiansen et al. (2021) [107] | 1,082 older adults aged 55 or older | Cognitive impairment | To analyse factors affecting mHealth technology use in relation to self-rated QoL among older adults with cognitive impairment | Cross-sectional research | Older adults | Mixed sample of adults aged above and under 60. |
| Duroseau et al. (2017) [108] | 109 participants with no age limits. | Parkinson’s disease | To assess patient opinions about technology-based tools, focusing on age-related differences | Cross-sectional research | Adults with Parkinson’s Disease | Mixed sample of adults aged above and under 60.  The study did not focus on technology targeting older people. |
| Gellis et al. (2012) [109] | 115 participants aged 65 and over. | Heart failure and COPD | The impact of a telehealth intervention on the health outcomes of older adults diagnosed with heart failure or COPD. | Randomised controlled trial | Older adults | The focus was not on barriers and facilitators to the adoption of digital health technology. |
| Johnson et al. (2014) [110] | 23 aged 56-93.  17 aged 63-89  6 aged 56-93. | COPD, hypertension, or diabetes | The focus was on describing the KSERA system, how it was validated and developed. | Mixed methods | Older adults | Mixed sample of adults aged above and under 60.  The focus was not on barriers and facilitators to the adoption of digital health technology. |
| Metting et al. (2018) [111] | 29 patients. No age range given.  There were no restrictions on the age of the participants. | COPD, Asthma | The focus was on patients’ perspectives on Patient Web Portals for disease self-management. | Qualitative (focus group) | Patients with COPD and asthma | Mixed sample of adults aged above and under 60.  The study did not focus on technology targeting older people. |
| Nancarrow et al. (2016) [112] | 200 participants with an age range of 48-98 | Multiple chronic disease.  Four participants didn’t have a chronic disease. | Ehealth benefits of using digital health technology. | Mixed methods (survey, interviews, focus groups) | Older adults | Mixed sample of adults aged above and under 60.  The focus was not on barriers and facilitators to the adoption of digital health technology. |
| Or et al. (2012) [113] | 50 participants aged 55 and over | Multiple chronic conditions | To evaluate a new computer-based self-management system interface for older adults with chronic diseases, using a paper prototype approach. | Mixed methods | Older adults | Mixed sample of adults aged above and under 60. |
| Price-Haywood et al. (2017) [114] | The criteria was age 50 and over. No age range provided.  There were 247 older adults. | Hypertension and diabetes | To investigate the relationship between e-health literacy and the use/non-use of a portal technology for self-care, and to identify barriers and facilitators to using the portal | Cross-sectional survey | Older adults | Mixed sample of adults aged above and under 60. |
| Reading Turchioe et al. (2020) [115] | 168 older adults  Age < 65 N=105 (62.5%)  65–74 N=46 (27.4%)  ≥ 75 N=17 (10.1%) | Heart failure | To evaluate the patient perceived usability of a mobile application for reporting health outcomes, and measuring differences in usability by age | Cross-sectional survey | Older adults | Mixed sample of adults aged above and under 60. |
| Robinson et al. (2020) [116] | 59 participants.  The age range was 49-89. | COPD | To investigate if age moderates multiple factors that influence the adoption of a physical activity device | Secondary data analysis | Older adults | Mixed sample of adults aged above and under 60. |
| Smaerup et al. (2016) [118] | 57 older adults aged 65 and over. | Vestibular dysfunction | To evaluate whether elderly patients with vestibular dysfunction are able to preserve physical functional level, quality of life, and reduction in dizziness, when assistive computer technology is used in comparison with printed instructions. | Randomized controlled trial | Older adults | The focus was not on barriers and facilitators to the adoption of digital health technology. |
| Zulfiqar et al. (2020) [120] | 36 participants aged 65 and older. | Multiple chronic conditions. | The focus was on evaluating the use of the MyPrediTM remote monitoring platform to detect symptoms in patients. | Quantitative (questionnaire) | Older adults | The focus was not on barriers and facilitators to using digital health technology. |
| Zulfiqar et al. (2021) [121] | 10 participants aged 65 and over | Diabetes | To evaluate the use of a remote monitoring platform and to experiment with this telemonitoring solution for diabetic elderly patients affected by the SARS-CoV-2 virus to prevent glycaemic disorder risk | Quantitative (questionnaire) | Older adults | The focus was not on barriers and facilitators to using digital health technology. |
